# The Role of Perceived Energy and Self-Beliefs for Physical Activity and Sports Activity of Patients With Multiple Sclerosis and Chronic Stroke

**DOI:** 10.3389/fpsyg.2020.570221

**Published:** 2021-01-28

**Authors:** Julia Schüler, Wanja Wolff, Julian Pfeifer, Romina Rihm, Jessica Reichel, Gerhard Rothacher, Christian Dettmers

**Affiliations:** ^1^Department of Sports Science, University of Konstanz, Konstanz, Germany; ^2^Educational Psychology Lab, University of Bern, Bern, Switzerland; ^3^Kliniken Schmieder, Konstanz, Germany

**Keywords:** physical activity, sport, multiple sclerosis, chronic stroke, fatigue, vitality, self-control, self-efficacy

## Abstract

Physical activity counteracts some of the negative consequences associated with chronic neurological diseases. Here, we describe the levels of physical activity (PA) and sports activity (Sport) in patients with multiple sclerosis (pMS, *n* = 59) and chronic stroke (pStroke, *n* = 67) and test compliance with the recommendation for health-promoting physical activity of the World-Health Organization (WHO). Secondly, we tested for differences between the groups of patients, and thirdly, we examined relationships between PA and Sport with psychological indicators of perceived energy (fatigue and vitality) and self-beliefs (self-efficacy and self-control). Psychological constructs were assessed with validated measures from different disciplines in Psychology. A statistical aim was to describe interpretations gained by (non-) parametric Bayesian and Null-Hypothesis-Significance Testing statistics (NHST) on the example of the conducted tests for differences and relationships. Descriptive analyses revealed that pMS and pStroke complied with recommendations of the WHO, but with large variance indicating that patient groups are not homogenous. Tests for differences showed that the PA difference between pMS and pStroke can be attributed to the higher proportion of women in the pMS sample as they engage more in household chores (important part of PA). Tests for relationships showed that for pStroke, vitality, self-control, and self-efficacy were positively related to the level of sports activity. Furthermore, pStroke who were sport active had lower fatigue and higher self-control and self-efficacy scores than sport inactive people. Although they address slightly different questions, the Bayesian and the NHST approach led to similar general conclusions.

## Introduction

Persons with chronic neurologic diseases such as multiple sclerosis (pMS) and post stroke impairments (pStroke) face significant declines in mobility and activities of daily living. This often compromises well-being and health-related quality of life. These undesirable consequences can be attenuated through physical activity, whereas physical inactivity may initiate a cycle of deconditioning and worsening of disease consequences (e.g., fatigue, immobility, social isolation). Excellent reviews and meta-analyses advocate the merits of exercise for well-being (e.g., reduced depressive symptoms, higher quality of life), and showed positive effects on cognitive functioning (Mandolesi et al., 2018) for adults with neurologic disorders such as multiple sclerosis (Pilutti et al., 2013; Adamson et al., 2015; Motl et al., 2017) and chronic stroke (Chen and Rimmer, 2011; Eng and Reime, 2014). For example, MS patients gait abnormalities and impairment (Comber et al., 2017; Liparoti et al., 2019) can be reduced by exercise (e.g., strength training, Mañago et al., 2019). Some authors even go so far to recommend “sports as medicine” (Dalgas et al., 2019). But the best medicine is superfluous if it is not taken. Indeed, MS patients “typically engage in low levels of health-promoting physical activity compared with adults from the general population, a fact which has not changed in the past 25 years despite growing evidence of the benefits of exercise (Motl et al., 2017, p. 848).”

It seems that—as is unfortunately also the case for the healthy general population—knowing what has to be done to improve one’s health is not enough (Schüler et al., 2019a). People often do not translate their knowledge and their intentions into action. Therefore, it is paramount to identify correlates of PA and Sport in chronic patients to better understand the psychological parameters that foster or inhibit an active lifestyle. This is not only crucial for theoretical reasons, but also to flag the key targets for behavioral intervention. In the present paper, we took an interdisciplinary perspective (psychology and neurorehabilitation) and within psychology, we used concepts from different research areas (coping research, motivation psychology, self-regulation research) to identify the theoretically most promising psychological correlates of PA and sport.

### Perceived Energy and Self-Beliefs

The choice of psychological correlates (out of the broad range of possible variables) was based on empirical and application-related pragmatic reasons: We chose variables that are well-known in current psychological research as predictors of physical activity and sport, but which are also closely related to the clinical symptoms of chronic patients. We therefore first chose variables assessing **perceived energy**. One variable is perceived ***fatigue***^1^ which is defined as an overwhelming feeling of physical and/or cognitive tiredness, along with a lack of energy (Krupp et al., 1988). Physical fatigue includes, for example, a perceived lack of physical power and muscle strength, as well as impaired physical stamina (Penner et al., 2009). Cognitive fatigue is often associated with, for example, difficulties in information processing, in maintaining attention, a decline of executive and cognitive functions, and loss of productivity, motivation, and drive (Berrios, 1990; Penner et al., 2009). Fatigue is a state of reduced functioning and well-being that interferes with the activities of daily living and strongly diminishes patients’ quality of life, and frequently, fatigue is regarded as the most distressing symptom by neurological patients (Comi et al., 2001; Bakshi, 2003).

Research shows that physical exercise counteracts fatigue and lack of energy in patients with chronic diseases (Motl et al., 2004; Stroud and Minahan, 2009; Larun et al., 2015; Van Vulpen et al., 2018). Meta-analyses, however, have often shown a more heterogeneous result pattern and identified moderators of the relationship between physical exercise and fatigue (Andreasen et al., 2011; Van Vulpen et al., 2018). Unclear relationships are partly traced back to patients’ lack of adherence to exercise regimes (Ammann et al., 2014) or by depression that can distort the relationship between PA and fatigue (Rietberg et al., 2011). We assume that a psychological construct that is on the other end of the energy spectrum might help in explaining some of the variance in PA and sports behavior of patients with chronic diseases: ***Subjective vitality*** is defined as “one’s conscious experience of possessing energy and aliveness” (Ryan and Frederick, 1997, p. 530) and therefore represents a positive variant of perceived energy. The motivation psychologists Ryan and Frederick (1997) referred to vitality as a specific psychological experience of feeling full of energy and possessing enthusiasm and spirit (p. 530). It is an indicator of well-being. The latter is defined as “a multi-faceted construct best described as a state of physical, psychological, and social health” (Pressman et al., 2013). Vitality is associated with psychological well-being (quality of life, low depression), and with somatic factors such as reduced physical symptoms and perceived body functioning in clinical and non-clinical samples (Ryan and Frederick, 1997; Salama-Younes et al., 2009; Rouse et al., 2014). Vitality is furthermore assumed to energize health-behavior (Niemiec et al., 2010) and is associated with physical activity and sports activity (Vlachopoulos and Karavani, 2009; Solberg et al., 2012; Özkara et al., 2017). Vitality and fatigue are positioned on opposing ends of the perceived energy spectrum that are likely to differentially explain variance in PA and sports behavior.

We call the second set of potential correlates of PA and Sport “**self-beliefs**,” by which we mean the perception (or belief) of the degree to which one is capable to deal successfully with difficulties and hindrances. ***Self-efficacy*** is defined as one’s confidence in the ability to execute specific actions required to achieve specific outcomes (Bandura, 1997). According to Social-Cognitive Theory (Bandura, 1986), self-efficacy has a decisive influence on the degree of challenge people strive for when setting goals and on the amount of effort they are willing to apply in order to attain their goals (Bandura, 1998). Among the plethora of psychological variables that have been linked to physical activity, self-efficacy represents one of the strongest and most consistent correlates (Sherwood and Jeffery, 2000; Higgins et al., 2014; Sheeran et al., 2016). The positive association between self-efficacy and physical activity has also been empirically demonstrated for patients with multiple sclerosis (Casey et al., 2016) and for patients after a stroke (Shamala et al., 2018). ***Self-control*** is the second self-belief we investigated. It is defined as the capacity to control one’s cognitive, emotional, and behavioral responses in order to bring them into line with the pursuit of long-term goals (Baumeister et al., 2007; De Ridder et al., 2018). This mainly includes prioritizing a goal (e.g., exercising regularly) by inhibiting impulses (resist the temptation to relax in front of the TV) and by initiating goal-directed actions (going to the gym) (Baumeister et al., 1994). A multitude of studies has shown that high self-control is conducive to various positive outcomes among them better short-term and long-term physical and mental health, interpersonal success, lower risk for substance dependence, higher socioeconomic status, higher income, and physical activity adherence (Tangney et al., 2004; Duckworth and Seligman, 2005; Moffitt et al., 2011; Hagger et al., 2019; Wolff et al., 2019a). It has even been shown, that MS patients with high self-control display a less steep increase in fatigue during a physically demanding task (Wolff et al., 2019b).

Not only due to an obvious similarity in content, but also due to a large proportion of studies insisting that self-control and self-efficacy are positively correlated (Graham and Bray, 2015; Yang et al., 2019), it seems justified to subsume self-efficacy and self-control under the header self-beliefs (as related perceptions that one is capable to deal successfully with difficulties and hindrances).

### Objectives of the Present Paper

The present paper’s objective is to analyze the correlates of physical activity and sports activity in people with chronic diseases. We chose a three-step **approach** to do so. First, we ***describe the physical activity and sports activity*** of two groups of patients (pMS and pStroke) and secondly, we ***tested for possible differences*** between these groups. We refer to physical activity (PA) as “any bodily movement produced by skeletal muscles that requires energy expenditure” (World Health Organization, 2008). Exercise or sports activity (Sport) is a subcategory of physical activity that is planned, structured, repetitive, and purposeful in the sense that the improvement or maintenance of one or more components of physical fitness is the objective (World Health Organization, 2020). PA is often done as part of playing, working, active transportation, household chores, and recreational activities. Whereas, these activities often require low to moderate expenditure of energy (in terms of metabolic equivalent, MET < 6), sports activity mainly includes activities of vigorous intensities (MET > 6) (Haskell, 2007). In the present research, we measured physical activity and sports activity of patients with multiple sclerosis (pMS) and chronic stroke patients (pStroke) and compare their activity level with WHO’s recommendations for health-promoting physical activity (World Health Organization, 2008). We then examined whether pMS and pStroke differ in their level of PA and Sport. Then, we ***tested for relationships*** between psychological indicators of perceived energy and self-beliefs with PA and Sport. Regarding perceived energy, we hypothesized that fatigue and vitality are related to levels of PA and Sport. Further, we expected a correlation between fatigue and vitality. As for self-beliefs, we tested the hypothesis that self-control and self-efficacy are associated with PA and sports activity, and with each other.

Furthermore, we intended to address a supplemental statistical topic. At first sight, ***testing for group differences***, and ***analyzing relationships*** is straightforward from a statistical point of view. But first impressions are deceptive, as the debate about how scientists draw conclusions from scientific data has shown (Francis, 2017). This so-called “statistical crisis” in science (Gelman and Loken, 2014) was mainly triggered by problems associated with ***Fisherian null hypothesis significance testing (NHST).*** Routinely, researchers have referred to the *p*-value (p: “the strength of the evidence against the hypothesis,” Fisher, 1958) to express their confidence in their data. However, using the p-value for inference testing has been massively criticized (Wagenmakers, 2007; Savalei and Dunn, 2015; Wagenmakers et al., 2018; Amrhein et al., 2019; McShane et al., 2019) (see also discussion part) and a plethora of statistical alternatives have been suggested (among them: focus on just discussing descriptive statistics, inference from confidence intervals, designing high power experiments, using criteria that quantify how good a statistical model fits the observed data such as AIC and BIC’s) (for a summary see Francis, 2017). A popular approach that relies on a different statistical framework is ***Bayesian statistics*** (Kass and Raftery, 1995; Berger, 2006; Wagenmakers, 2007; Wagenmakers et al., 2018). Bayes-Factors are defined as the ratio of two probabilities, namely, the probability of the data when the null-hypothesis is true and the probability of the data when the null-hypothesis is false. In the following analyses, we reported the Bayes-Factor (BF) BF_01_. It expresses the likelihood of the null model (in our analyses: no difference, no relationships) relative to an alternative hypothesis (in our analyses: significant differences, significant relationships). In statistical terms, BF_01_ is the likelihood of no-significant differences/relationships (H0) divided by the likelihood of significant differences/relationships (H1). A BF_01_ of 2.00, for example, suggests that the data are twice as likely to be observed under the null hypothesis. Taking the inverse, the data are half as likely under the alternative hypothesis. Thus, a Bayesian approach essentially addresses a different question than Fisherian NSHT and this yields a different type of answer (e.g., The ability to quantify evidence for a hypothesis and not merely against it.) and this might be closer to what a researcher is actually trying to answer (for an overview see, Wagenmakers et al., 2018). Above all, it avoids dichotomous thinking by not drawing conclusions from a relatively arbitrary threshold (e.g., *p* < 0.05) (for an overview see Wagenmakers et al., 2018), and allows quantifying evidence without needing to know the intention with which the data were collected (Wagenmakers, 2007).

Amidst the discussion about the pros and cons of different statistics, Francis (2017) has summarized equivalent statistics and concluded that statistics might have legitimate differences, but that they are nevertheless closely related because they drive their properties from the very same information in a set of data. He stated—referring to the example of an independent two-sample *T*-test with equal population variances—that “many of the various statistics are mathematically equivalent representations of the information from the data set, and their corresponding analysis methods differ only in how they interpret that information” (p. 1525). Rejecting the idea that some statistical methods are principally better than others, he concluded that scientists should choose an analysis method that is appropriate for their research question and data sets.

Appropriateness of analyses also refers to the distribution of variables. Whereas in the last decade, ***Bayesian parametric tests*** have often been used and discussed, ***Bayesian non-parametric*** tests have been utilized less often (for an exception see Yuan and Johnson, 2008; Ghosal and Vaart, 2017; Griffin et al., 2017; Hai, 2017). This is important, however, because some variables are known to be not normally distributed. This applies to the core constructs of our research “PA” and “Sport” and their antecedents and consequences (Rebar et al., 2019). Often, they are extremely positively or negatively skewed (Dilorio, 2006). Because unmet model assumptions carry the risk of misinterpreting tests (Rebar et al., 2019), using non-parametric tests and—pursuing our argument from above—using Bayesian non-parametric tests could be an appropriate approach to analyze PA and Sport data.

Our data seem to be well suited to comparatively highlight non-parametric Bayesian and Null-hypothesis Significance Testing (NHST) statistics. To do so, we analyzed our data twice. First with a Bayesian and then with the NHST approach. Table 1 shows which Bayesian and NHST-statistics we used for testing differences and for testing relationships of variables that are normally and not normally distributed, respectively (what has to be tested in advance). Table 1 also summarized interpretations gained from both approaches that we will outline in the results part.

**TABLE 1:** Statistical tests used to address our research aims (upper part) and summary of significant results (lower part).

Statistical tests				
	Non-parametric tests		Parametric tests	
	Bayes	NHST	Bayes	NHST
Tests for differences	Bayesian		Bayesian	
	Independent	Independent	Independent	Independent
	Samples *T*-tests	Samples *T*-test	Samples *T*-test	Samples *T*-test
	Mann-Whitney	Mann-Whitney	Student	Student
Tests for relationships	Bayesian		Bayesian	
	Correlation	Correlation	Correlation	Correlation
	Kendall’s tau	Kendall’s tau	Pearson	Pearson

**Summary of results**				

	**Non-parametric tests**		**Parametric tests**	
	**Bayes^a^**	**NHST^b^**	**Bayes^a^**	**NHST^b^**

Tests for differences	Weak	Significant	Weak	Significant
	Weak	Significant	Weak	High
	Weak	Significant	–	–
	Weak	Significant	–	–
	Weak	High	–	–
	Weak	High	–	–
	Strong	Very high	–	–
Tests for relationships	Weak	Marginal	Positive	High
	Weak	Marginal	Positive	Very high
	Weak	Significant	–	–
	Weak	High	–	–
	Positive	High	–	–
	Positive	High	–	–
	Strong	Very high	–	–
	Strong	Very high	–	–
	Strong	Very high	–	–

*^*a*^Bayes: weak, positive, and strong evidence for differences/relationships. ^*b*^NHST: marginal, significant, high, and very high significance of effects.*

Several authors suggested using certain terminologies that help to interpret Bayes factors (Raftery, 1995; Wetzels et al., 2011). We follow Raftery’s guidelines to interpret Bayes-Factors as evidence for the alternative hypotheses by describing Bayes-Factors (BF_01_) of 1.0–0.33 as “weak,” BF_01_s of 0.33–0.05 as “positive,” BF_01_s of 0.05–0.0067 as “strong,” and BF_01_s < 0.0067 as “very strong” (Raftery, 1995). For NSHT, we used the terms “significant effect” for *p* < 0.05, “highly significant effect” for *p* < 0.01, and “very highly significant effect” for *p* < 0.001. We are aware (and we will discuss it later) that many scientists recommend to abandon the term “statistical significant” (Hurlbert et al., 2019; Wasserstein et al., 2019), but would like to use it here to highlight how commonly used NHST and its terminology might differ from Bayesian statistics and terminology. Please note that we did neither hypothesize differences between both approaches from the beginning nor that our data set is special compared to previously collected data. Thus, from a statistical perspective, our aim is to describe these different statistical approaches, highlight the differences in interpretations they allow, and depict how the proposed interpretational terms might categorize the same findings differently.

## Materials and Methods

### Participants and Procedure

A total of 124 participants were recruited from two Clinics for Neurological Rehabilitation in Constance and Gailingen, Germany. Sixty-seven stroke patients (31 women) with a mean age of 55.28 years (*SD* = 11.85, range: 26–81) and 59 MS patients (41 women) with a mean age of 50.80 years (*SD* = 10.81; range: 25–84) took part in the study. Time since diagnosis was *M* = 16.69 years (*SD* = 9.82) for multiple sclerosis and *M* = 5.50 years (*SD* = 5.08) for stroke patients. One-third of MS patients (32.2%) and 24.6% of stroke patients work full-time and 27.1% (MS) and 15.4% (stroke) work part-time. 3.0% of MS patients and 3.5% of stroke patients were housewives and househusbands repo. 8.5% of MS patients and 15.4% of stroke patients were age retired. 27.1% of MS and 16.9% of stroke patients were retired due to incapacity to work. The others reported being on sick leave.

Data were collected by a self-administered questionnaire. It started with an informed consent form, followed by asking patients to indicate their gender, age, the time since diagnosis, whether they were diagnosed with multiple sclerosis or stroke, and their professional activities. Then the participant filled in the measures described below and finished the questionnaire within 30 and 45 min and were fully debriefed. The study was conducted in accordance with the declarations of Helsinki (World Medical Association, 2013).

### Measure

***Physical activity and sports activity*** were measured using the *Physical Activity, Exercise, and Sport Questionnaire* (Bewegungs- und Sportaktivität-Fragebogen, BSA, Fuchs et al., 2015). It is an economic and clearly structured instrument based on FITT technology (Frequency-Intensity-Time-Type, see Sallis and Owen, 1999). To reliably measure physical activity, its frequency, its duration, its intensity, and the type of sports activity has to be considered (Fuchs et al., 2015). The intensity (as part of FITT) was not directly assessed in the BSA but estimated from the usual level of the required energy for a task (metabolic equivalents of tasks, see, Kohl and Murray, 2012). The items for physical activity in the BSA (Fuchs et al., 2015) (first part of BSA, see below) can be classified as moderately intensive (METs between 3.0 and 6.0, see Kohl and Murray, 2012). The intensity of sports activities (second part of BSA, see below) listed by the patients could be classified as vigorous (METs > 6) (e.g., tennis, jogging), although some activities might be classified as moderate (e.g., bicycling light effort).

In the first part of the BSA, participants indicated the frequency (how often in the last 4 weeks) and time (minutes per day) of eight physical activities (walk to work, go shopping on foot, go to work by bicycle, cycling for the purpose of transportation, gardening, strenuous housework, physically strenuous care work, such as childcare or nursing the sick, physical exertion at work). Then participants rated whether they had been sport active during the last 4 weeks. If they answered yes, they were asked to indicate the type of sport, frequency, and time. If they answered no, they could skip the following block of questions. Please note, that the original BSA has a third part that we omitted from this study because it solely measures PA related to work.

To assess ***perceived energy*** the *Fatigue Scale for Motor and Cognitive Functions (FSMC)* (Penner et al., 2009) was used that captures cognitive fatigue (e.g., “When I am experiencing episodes of exhaustion, I lose concentration considerably quicker than I used to”) and motor fatigue (e.g., “When I am experiencing episodes of exhaustion, my movements become noticeably clumsier and less coordinated”) with 10 items each. For the present research question, we averaged all items and used an overall fatigue score. Participants indicate their agreement to the statements using a 5-point Likert scale (1: does not apply at all; 5: applies completely). Internal consistency in the present study was high with Cronbach’s Alpha = 0.98. The *Vitality scale* (Ryan and Frederick, 1997) assesses individual differences in felt vitality (as a kind of trait measure) with seven items (e.g., “I feel alive and vital,” “I have energy and spirit,” “I feel energized.”). Participants indicate the degree to which the statement is true for them in general in their life using a 7-point rating scale (1: not at all true; 7: very true). Cronbach’s alpha in this study was 0.85.

*Self-control*, as one aspect of ***self-beliefs***, was assessed using a German version of the brief version of the *Self-Control Scale* by Tangney et al. (2004) (German version: Bertrams and Dickhäuser, 2009). Using a 5-point rating scale (1:“completely incorrect”; 5: “completely correct”) participants indicated their agreement to 13 items (e.g., “I am good in resisting temptations,” “Other people would say that I have iron self-discipline.”). Cronbach’s alpha was 0.72. *Self-efficacy* was assessed using a sport-specific scale proposed by Fuchs and Schwarzer (1994). Participants read the beginning of the sentence “I am sure that I can still carry out a planned sporting activity even if.” and responded to the following 12 statements (e.g., “… I am tired,” “…I am feeling low,” “… the weather is bad”) using a 7-point rating scale (1: “I am not at all sure”; 7: “I am quite sure”). Herewith, task self-efficacy rather than general self-efficacy was assessed (Bandura, 1997). The scale was reliable with Cronbach’s alpha = 0.84.

Data analyses were conducted using the freely available open- source software JASP (JASP Team, 2019, Version 0.11.1, Computer software).

## Results

### Testing for Normal Distribution

**FIGURE 1:**
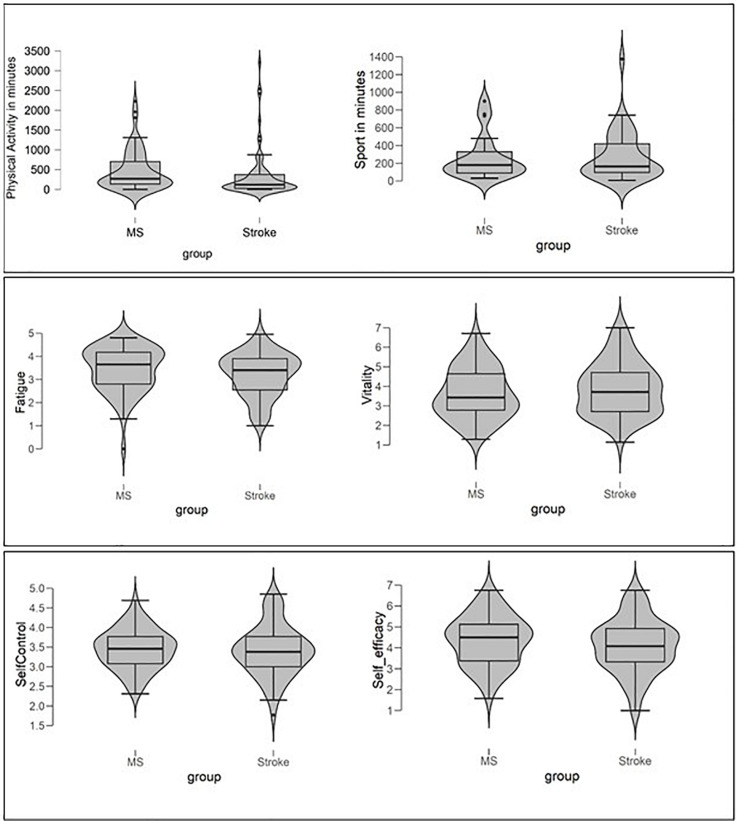
Violin plots and Box-Plots for physical activity and sports activity (upper part of figure), perceived energy (fatigue, vitality), and self-believes (self-control, self-efficacy) of pMS and pStroke.

The boxplots displayed in Figure 1 allow a visual inspection of the distribution of PA, Sport, perceived energy, and self-beliefs. Self-efficacy, self-control, and vitality look approximately normally distributed, whereas all other variables are clearly not normally distributed. As in previous studies (e.g., Dilorio, 2006), especially PA and Sport are extremely skewed. Additional Shapiro-Wilk tests, which are summarized in Table 2, confirmed these conclusions from visual inspection.

**TABLE 2:** Descriptive Statistics and results of tests for normal distribution separately for pMS and pStroke.

	Vitality		Fatigue		Self-control		Self-efficacy		PA		Sport	
	pMS	pStroke	pMS	pStroke	pMS	pStroke	pMS	pStroke	pMS	pStroke	pMS	pStroke
Valid	59	65	59	65	59	65	59	65	59	65	37	34
*M*	3.642	3.892	3.431	3.205	3.448	3.407	4.350	4.097	472.292	358.444	260.608	278.408
*SD*	1.287	1.347	0.973	0.927	0.507	0.647	1.178	1.287	514.671	632.259	247.266	284.023
Shapiro-Wilk (W)	0.973	0.970	0.935	0.958	0.991	0.984	0.988	0.984	0.815	0.591	0.780	0.789
*p*-value of W	0.202	0.109	0.003	0.028	0.953	0.581	0.844	0.580	<0.001	<0.001	<0.001	<0.001
Minimum	1.290	1.140	0.000	1.000	2.310	1.770	1.580	1.000	0.000	0.000	30.000	7.500
Maximum	6.710	7.000	4.800	4.950	4.690	4.850	6.750	6.750	2227.500	3210.000	900.000	1375.000

*Variables that are normally distributed are underlined.*

### Preliminary Analyses

As preliminary analyses, we tested whether pMS and pStroke differed in demographic and clinical variables that were unrelated to the hypotheses. Participants’ age and time of diagnosis were not normally distributed in the subsample of pStroke (Shapiro-Wilk test: *W* = 0.96, *p* = 0.02 for age, *W* = 0.81, *p* < 0.001 for time since diagnosis), and therefore Bayesian Mann-Whitney U-Tests were used. As displayed in the upper part of Table 3, the Bayesian analyses indicated weak evidence for the hypothesis that pMS and pStroke differ in their age. The NHST analysis was highly significant, indicating that the H0 should be rejected. Patient groups also differed in their time since diagnosis (Bayes: strong evidence for effect, NHST: highly significant effect).

**TABLE 3:** Testing for differences between pMS and pStroke in control variables, in physical activity and sports activity, in perceived energy, and in self-beliefs using Bayesian and NHST Mann-Whitney-*U* Tests and Student Tests, respectively^a^.

	Bayesian		NHST		pMS		pStroke	
	*BF*_01_	*W*	*p*	*W*	*M*	*SD*	*M*	*SD*
Age	0.462*	2.463	0.006**	1.372	50.80	10.81	55.28	11.85
Time diagnosis	6.85e-***	593.5	0.0001***	3.241	16.69	9.82	5.50	5.08
PA	0.784*	1408.0	0.011*	2.427	472.29	514.67	358.4	632.26
Sport	4.283	622.5	0.945	635.5	260.60	247.3	278.4	7.50
Fatigue	1.786	1612.5	0.128	2222.5	3.43	0.97	3.29	0.93
Vitality	3.157	(0.006)	0.294	(−1.053)	3.64	1.29	3.89	1.35
Self-control	4.867	(0.027)	0.697	(0.390)	3.45	0.51	3.41	0.65
Self-efficacy	2.904	(0.004)	0.257	(1.138)	4.35	1.18	4.10	1.29

*BF_01_ < 1.0, **BF_01_ < 0.30, ***BF_01_ < 0.01, *p < 0.05, **p < 0.01, ***p < 0.001. ^*a*^For underlined variables Student T-test were computed (Bayes: scores in brackets mean error%; NHST: scores in brackets mean T-values, df = 1, 122). For all other comparisons, Bayesian Mann-Whitney-U Tests were used (Results were based on data augmentation algorithm with 5 chains of 1,000 iterations).

Additional preliminary analyses tested whether participants’ age or gender are related to the dependent variables PA and Sport. Bayesian Mann-Whitney *U*-Test showed positive evidence for differences between men and women in their sports activity (BF_01_ = 0.547, W = 817.0) (NHST Mann-Whitney *U* Test: *W* = 817, *p* = 0.029) with higher scores for men (*M* = 360.7, *SD* = 325.1) than for women (*M* = 189.6, *SD* = 161.9), whereas they do not differ in their physical activity (BF_01_ = 3.379, *W* = 1672, men: *M* = 398.2, *SD* = 603.4, women: *M* = 423.0, *SD* = 566.2) (NHST: W = 1671, *p* = 0.310). Bayesian correlational analyses (Kendall’s tau-b) showed weak evidence for the interpretation that age was negatively related to PA (Kendall’s tau = −0.163, BF_01_ = 0.240), whereas NHST revealed a highly significant correlation (*p* = 0.009). Age was unrelated to Sport (Kendall’s tau = −0.073, BF_01_ = 4.353) (*p* = 0.383). Duration of diagnosis was neither correlated with Sport (Kendall’s tau = −0.085, BF_01_ = 3.762) (*p* = 0.303), nor with PA (Kendall’s tau = −0.015, BF_01_ = 8.260) (*p* = 0.805).

### Describing PA and Sport

Whereas all participants indicated at least some amount of PA, only 39 pMS (66.1%) and 34 pStroke (52.3%) claimed to do sport at all (most frequently mentioned: Nordic Walking, cycling, cross-trainer). Thus, in the following analyses, the variable Sport is based on reduced sample size.

The Violin and Box-Whisker-Plots on the upper left part of Figure 1 illustrate the amount of PA and Sport for pMS and pStroke graphically, and Table 3 shows exact means and standard deviations. All distributions are left-skewed. Comparing the violine shapes of the distributions shows that for both groups a small number of extreme values are pulling the means for PA and Sport up, but that this trend is more pronounced for pStroke (outliers are marked by dots, pMS: 5.08%, pStroke: 9.32%).

### Testing for Differences Between pMS and pStroke

As described in detail in Table 3, Bayesian Mann-Whitney *U*-Tests supported the null-model (NHST: no rejection of H0), indicating no differences between pMS and pStroke for sports activity. For PA, however, the data supported weak evidence for the alternative model indicating evidence for differences between pMS and pStroke. In accordance, the NHST variant of the Mann-Whitney *U*-Test was significant, thereby rejecting the H0. In supplemental analyses, we investigated in more detail what type of PA accounts for the significant difference in the sum score of PA. Considering the single items “walking to work,” “going shopping on foot,” “going to work by bicycle,” “cycling for the purpose of transportation,” “gardening,” “physically strenuous care work,” and “physical exertion at work” in separate analyses, yielded no significant differences between pMS and pStroke. Patient groups only differed in regard to the BSA item assessing “strenuous housework” (Bayes: BF_01_ = 0.631, *W* = 1468.5, *R*^2^ = 1.003; NHST: *W* = 2366.5, *p* = 0.02) with pMS engaging on average twice as long in strenuous housework than pStroke (pMS: *M* = minutes, *SD* = 327.89; pStroke: *M* = 102.29 min, *SD* = 212.12). Also men and women differed significantly in “strenuous housework” (BF_01_ = 0.503, *W* = 2411.5, *R*^2^ = 1.018) (*W* = 1332.5, *p* = 0.005) with higher scores for women (*M* = 202.66, *SD* = 311.91) than for men (*M* = 92.55, *SD* = 209.72). Because the group of pMS contains more women (56.94%, *n* = 41) than the group of pSTroke (43.06%, *n* = 31) [χ^2^(1) = 6.04, *p* = 0.041], we considered participants’ gender as a random factor in a Bayesian ANCOVA and added it to the null model. In this analysis the data do not further support significant differences between pMS and pStroke in regard to “strenuous housework” [BF_01_ = 1.01, error% = 0.98; NHST: *F*(1, 120) = 3.167, *p* = 0.08].

A Bayesian ANCOVA controlling for differences between men and women in sports activity (see preliminary analyses), revealed no support for differences between pMS and pStroke in sports activity (BF_01_ = 3.861, error% = 1.823) [NHST: *F*(1, 67) = 0.143, *p* = 0.706].

Supplemental analyses found no evidence in the data for differences between pMS and pStroke in variables representing perceived energy (fatigue, vitality) and self-beliefs (self-efficacy, self-control) (see lower part of Table 3).

### Testing Relationships Between PA, Sport, and Psychological Variables

Bayesian and Non-Bayesian correlational analyses (Kendall’s tau, Pearson) are displayed separately for pMS and pStroke in Table 4. The frames in the upper part of Table 4 indicate correlations within ***perceived energy*** variables and within ***perceived self-belief*** variables, respectively, for which we formulated hypotheses. Data revealed very strong evidence that for pMS and for pStroke the ***perceived energy*** variables, “vitality” and “fatigue” are negatively related (Bayes: strong evidence, NHST: highly significant effect). The ***self-belief*** variables self-control and self-efficacy were positively related but only for the group of stroke patients (Bayes: strong evidence, NHST: highly significant effect). Also between ***self-belief*** variable and ***perceived energy*** scores, relevant correlations were found in both groups of patients: Self-control was negatively correlated with fatigue (Bayes: positive evidence, NHST: highly significant effects) and positively related with vitality (Bayes: positive/strong evidence, NHST: very highly significance). Self-efficacy was also related to perceived energy scores, but only for the group of pStroke: A negative relationship between self-efficacy and fatigue (Bayes: weak evidence, NHST: marginal effect) and a positive relationship with vitality (Bayes: positive evidence, NHST: highly significant effect) was found.

**TABLE 4:** Correlation analyses (Kendall’s tau, two-tailed) based on Bayesian statistics and NHST separated for pMS and pStroke.

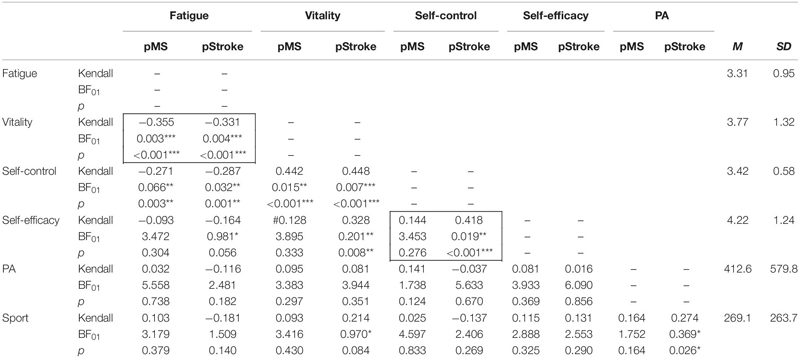

Correlations within perceived energy/self-belief variables are framed. Means and standard deviations refer to the full sample. *BF_01_ < 1.0, **BF_01_ < 0.30, ***BF_01_ < 0.01, *p < 0.05, **p < 0.01, ***p < 0.001. Underlined variables: Pearson correlations were computed when both variables were normally distributed.

The lower part of Table 4 shows the relationships between PA and Sport and perceived energy and self-beliefs. In contrast to our hypotheses, PA, and Sport were unrelated to all these psychological variables. One exception—that, however, only applies to the group of pStroke—was a positive relationship between vitality and Sport (Bayes: weak effect; NHST: marginal effect). PA and sport were positively related to each other (Bayes: weak evidence, NHST: significant effect).

In further analyses, we considered that a considerable proportion of patients did not exercise at all (sport inactive, MS: 33.9%, Stroke: 47.7%). Analyses testing for differences between sport active and sport inactive people are displayed in Table 5. Only in the group of pStroke, sport active and sport inactive people differed in fatigue, self-control, and self-efficacy with higher scores for sport active people.

**TABLE 5:** Bayesian and NHST-Mann-Whitney-*U* Tests or Student tests analyzing differences between sport active and sport inactive people in perceived energy (fatigue, vitality) and self-beliefs (self-efficacy, self-control).

		pMS						pStroke					
				Bayesian		NHST				Bayesian		NHST	
		*M*	*SD*	*W*	BF_01_	*W*	*p*	*M*	*SD*	*W*	BF_01_	*W*	*p*
Fatigue	Active	3.46	0.75	420.0	3.576	360.0	0.636	2.92	0.96	706.5	0.394*	347.5	0.019*
	Inactive	3.37	1.32					3.51	0.79				
Vitality	Active	3.83	1.32	(0.007)	1.266	(1.602)	0.115	4.15	1.41	(0.005)	1.268	(1.642)	0.106
	Inactive	3.27	1.17					3.61	1.23				
Self-control	Active	3.47	0.53	(0.009)	1.438	(−0.282)	0.779	3.22	0.62	(0.004)	0.479*	(2.251)	0.028*
	Inactive	3.43	0.50					3.57	0.64				
Self-efficacy	Active	4.39	1.16	(0.014)	3.422	(0.368)	0.715	1.33	0.23	(7.84e-4)	0.113*	(2.945)	0.005**
	Inactive	4.27	1.23					1.07	0.19				

*BF_01_ < 1.0, **BF_01_ < 0.30, *p < 0.05, **p < 0.01. For underlined variables Student T-test were computed (Bayes: scores in brackets mean error%; NHST: scores in brackets mean T-values, df for pMS = 57, df for pstroke = 63). For all other comparisons, Bayesian or NHST Mann-Whitney-U Tests were used. For Bayesian tests results were based on data augmentation algorithm with 5 chains of 1,000 iterations).

## Discussion

Our primary aims were to describe PA and Sport of pMS and pStroke, to test differences between groups of patients, and to test for relationships with perceived energy (fatigue and vitality) and self-beliefs (self-control, self-efficacy).

### Describing Physical Activity and Sports Activity

Using the BSA questionnaire (Fuchs et al., 2015) allowed us to analyze the levels of PA, to investigate how many participants are sport active and sport inactive, respectively, and to examine the level of sports activity of those who are sport active. The point of reference that we pit the PA data against, are the recommendations of the World Health Organization (WHO, World Health Organization, 2008). The WHO recommends at least 150 min of moderate-intensity physical activity or at least 75 min of vigorous-intensity physical activity throughout the week. With 472.3 min (pMS) and 358.4 min (pStroke) of physical activity per week, both groups of patients seem to clearly fulfill and even exceeded the recommendations of the WHO for PA (150 min). This was confirmed by One-Sample-*T*-tests (Bayesian One-Sample *T*-test, pMS: BF_01_ = 1.206e-4, error% = 3.580e-10, pStroke: BF_01_ = 0.292, error% = 2.109e-6). Also, people who are sport active (58.9%) (full sample: *M* = 269.1 min, *SD* = 263.7) perfectly act upon the WHO’s recommendations to do a least 75 min of vigorous-intensity physical activity.

The surprisingly high level of physical activity in our sample might be traced back to people’s general tendencies to wrongly estimate (e.g., overestimate or underestimate) the amount and level of physical activity when indicating it in questionnaires (Martins et al., 2017). Only low to moderate correlations have been found with objectively assessed (e.g., accelerometry, doubly labeled water, direct observations, calorimetry, physiological markers) physical activity (Prince et al., 2008; Silsbury et al., 2015). This also applies to the BSA for which low-to-moderate correlations with VO_2_ max, and endurance performance have been reported (Fuchs et al., 2015). Although direct measures of physical activity might provide more accurate assessments of physiological parameters that correspond to the physical activity levels, self-reports are less expensive and less time consuming and are easier to implement in clinical settings (see also, Ainsworth et al., 2015). One important criterion that we used to evaluate the appropriateness of a questionnaire of physical activity is the clinical utility of this tool. Is it, for example, easy to administer (no special expertise required by the experimenter)? Is it short and easy to understand so that it is appropriate for people suffering from fatigue? Does it allow to identify specific forms of physical activity that can be used for concrete recommendations (e.g., spend more time on the bicycle or with strenuous housework)? These questions can be positively answered for the BSA.

Rather than using the WHO recommendations as a point of reference, a more meaningful comparison of the physical activity level of our sample might be with other studies that used the BSA (for a summary see, Fuchs et al., 2015). Patients of our study reported to be less physical active than orthopedic patients of a rehabilitation clinic (PA: *M* = 506.25 min, *SD* = 504.79, *M*age = 51.0 years) (Fuchs et al., 2011), but more physically active than overweight and obese people (PA: *M* = 342.05, *SD* = 261.21, *M*age = 48 years) (Gerber et al., 2010). Also, these groups of people clearly exceeded the recommendations of the WHO. It is also worth noting that BSA’s physical activity levels of male clerks (PA: *M* = 328.1, *SD* = 199.9, *M*age = 45.7 years) (Fuchs et al., 2015), and young and healthy women (aged between 18 and 28, PA: *M* = 390.90 min, *SD* = 261.36) (Klaperski et al., 2013) are lower than for patients in rehabilitation clinics. It remains to be investigated in future studies whether the general rehabilitation setting itself (e.g., recommendations by doctors and physiotherapists; increased focus on health issues and healthy lifestyle) promotes physical activity. Future studies might also include an age-matched healthy control group so that the comparison between patient groups and healthy people can answer related and highly relevant research questions.

Summing up, on first sight, the average level of PA looks quiet good. A second look at the data, however, showed large variances in our groups of patients (see shapes of violins in Figure 1), indicated that some people clearly fulfill or even exceed the PA and sport recommendations, whereas others fail to do so. Expressed in numbers, 54 out of 124 patients (43.5%) are less than 150 min physically active. In sum, according to our data, pMS and pStroke did not differ from non-chronically ill people with regard to PA and Sport.

The distributions additionally show that all outliers lie above the mean, indicating that some pMS and pStroke show exemplary PA and sport, despite their chronic diseases. It could be a promising approach to gain a larger sample of these “extreme” (extreme in terms of PA and Sport) people and compare them with patients with lower PA scores and/or with sport inactive people to figure out in which psychological (e.g., personality characteristics, previous experiences with PA and Sport), physiological (e.g., pain, status of health), and social characteristics (e.g, social support) they differ from each other.

### Testing Differences Between pMS and pStroke

As we expected based on previous studies (Rebar et al., 2019), neither PA nor sports activity was normally distributed. The same applies to fatigue, whereas vitality, self-control, and self-efficacy were normally distributed. We, therefore, used parametric and non-parametric tests of differences, respectively (see Table 1). We found that pMS were more physically active than pStroke, but groups did not differ in their sports activity. The difference in PA, however, could be traced back to the single variable “strenuous housework” and disappeared when we controlled for participants’ gender. On the one hand, this shows that it is worthwhile to carry out more differentiated analyses before drawing conclusions. The group of pMS is not *per se* physically more active than the group of pStroke, but contains more women who in turn do more household chores. On the other hand, this finding draws attention to the fact that the often unloved household tasks (in addition to the cleanliness achieved) have an additional positive effect on health. Brooks et al. (2004), for example, analyzed energy expenditure during self-paced household tasks such as sweeping, window cleaning, vacuuming and mowing by using sophisticated indirect measures (e.g., heart rate, respiratory frequency, Computer Science Applications, hip and wrist movement counts) in addition to self-reports (Borg rating of perceived exertion) and found METs of 3.0 or higher for these activities (Brooks et al., 2004, Please note that Brook’s sample exclusively consisted of women). This data suggests that the aforementioned household chores can contribute to the “30 min a day rule” of moderate-intensity activity required to confer health benefits. Educating especially male patients with chronic diseases about this could make an important contribution to the promotion of PA and health.

What has to be critically mentioned is that in our sample groups of pMS and pStroke differed from each other in their mean age, in the duration of diagnosis and in the proportion of men and women. Although analyses controlling for these variables revealed that they did not affect the reported results, future studies addressing similar research questions could pay more attention to equality of groups. In order to get even deeper insight into PA and sport activity of pMS and pStroke, more clinical characteristics of the patients could be considered. For example, knowing patients’ mental states (e.g., dementia, depression, anxiety), and severity of disease might give an even more differentiated view on patients PA and sport activity.

### Testing Relationships of PA and Sport With Perceived Energy and Self-Beliefs

In disconcordance with our hypotheses, neither the perceived energy variables nor the self-beliefs variables were correlated with PA and Sport. It seems as if the *level* of PA and Sport is unrelated to these psychological variables, whereas these variables are relevant for the fact of *whether or not* a person is sport active or inactive (at least for pStroke). For the group of pStroke, people who are sport active reported lower fatigue, higher self-control, and higher self-efficacy than sport inactive people.

In the present study, we consequently used the term “correlates” of PA and Sport to state clearly that our cross-sectional design prohibits the drawing of causal conclusions. Hereby we have avoided the question of causality, which is important but also tricky. Whether, for example, sports activity leads to feelings of vitality, self-control and self-efficacy, or whether vitality, self-control and self-efficacy foster sports activity is unclear. However, as already mentioned in the introduction, evidence for a positive causal relationship comes from existing RCT-based studies showing that exercising leads to lower fatigue (Ammann et al., 2014) and higher self-efficacy (Shamala et al., 2018). Also epidemiological research that uses other criteria for evaluating the strength of evidence (i.e., strength of relationship, temporal sequence, consistency, dose-response and biological plausibility) has suggested a strong, consistent, temporally appropriate dose-response relationship between physical activity and feelings of energy and fatigue (Puetz et al., 2006). However, as far as the authors know, there are no RCT studies or epidemiological research that show that exercise increases self-control.

Longitudinal cross-lagged panel designs are needed to test for the even more complex assumption that there are not only two simple directions of causality, but also mutual influences. Fatigue, for instance, could lead to low levels of PA which in turn leads to more fatigue. Or the low level of self-efficacy might lead to low levels of sport which in turn further diminishes self-efficacy (Biddle and Mutrie, 2007). The good thing about such vicious circles is, however, that they can be broken at two points. First, in accordance with Dalgas et al. (2019)’s term of “sport as medicine,” physical activity and sport lessons should be prescribed by physicians. Second, self-control can be enhanced by training (Friese et al., 2017; Naetes et al., 2020) and self-efficacy for PA can be strengthened by behavior change techniques (Ashford et al., 2010; Olander et al., 2013). As true for healthy people, changing perceived energy and self-beliefs is undoubtfully a good start, but probably still not enough. Volitional strategies such as concrete planning strategies (Gollwitzer and Sheeran, 2006) are additionally needed to put PA and Sport intentions of people with chronic disorders into action (Schüler et al., 2019b). To avoid that sport becomes another burden in an already stressful life (of chronically ill people and of healthy people as well), a sports activity should be chosen that fits to a person’s individual needs and interests (Sheldon and Elliot, 1999) so that the incentives inherent in the activity (enjoying sport context, fun, positive emotions) support the outcome related incentives (better health) and thereby secure behavior over a longer period of time (for activity and outcome-related incentives see, Rheinberg, 1989; Rheinberg and Engeser, 2010; Schüler et al., 2019b).

### Bayesian and NHST Statistics

Our statistical aim was to report the results and interpretations from different statistical approaches. Depending on the variables’ distributions, we used parametric or non-parametric tests to examine ***differences*** between groups of patients and ***relationships*** of PA and Sport with psychological variables. As summarized in Table 1, the result pattern for Bayesian and NHST mainly looks similar. Out of 26 tests for differences (reported in Tables 3, 5 and in the text), Bayesian and NHST statistics revealed evidence for alternative hypotheses and for significant differences, respectively, in the same nine tests. Bayesian statistics seemed a bit more conservative in interpreting the data by indicating three effects as “weak evidence,” that NHST would label as “highly significant.”

Out of the tests for relationships (30 reported in Table 4, five reported in text), Bayesian and NHST again identified the same eleven relationships as relevant. In two cases, NHST was a bit more conservative by indicating relationships as “marginal,” for which Bayesian statistics found “weak evidence” for an effect. In one case NHST labeled a relationship as “highly significant” for which Bayesian statistics only found weak evidence in the data. Another relationship was indicated as “weak evidence for alternative hypothesis” in Bayesian statistics, but as a “highly significant effect” in NHST.

Critics of the NHST approach may rightly argue that using the term “statistical significant effect”—as we did in this paper—causes severe problems and may recommend in general “don’t say statistically significant” (Hurlbert et al., 2019; Wasserstein et al., 2019). Reliance on thresholds (*p* < 0.05) is misleading because *p* < 0.05 and *p* > 0.05 do not necessarily mean that an effect exists or does not exist, respectively. Describing the results and their interpretation of Bayesian and NHST as we did, might pose further problems. The way we listed results in Table 1 might (mis)lead the reader to the assumption that we “compared” interpretations from Bayes and NHST statistics. But of course, the terms used in the literature to describe results (e.g., NHST: highly significant effects, Bayes: positive evidence) are not directly comparable—The Bayesian terms were explicitly designed to avoid terms of NHST. Even from a linguistical perspective, the terms are misleading with Bayesian statistics using less strong adjectives to describe evidence (e.g., “weak,” “positive,” “strong“) than NHST that uses stronger adjectives to describe effects (“significant,” “highly significant,” “very highly significant.” Purely linguistically, that might trigger different associations in readers when evaluating the relevance of data. Further, Bayesian statistics and NHST are different statistical frameworks, that ask different questions from the data, and that in turn also yields a different type of answer. Thus, in addition to the use of different terminology for classifying results, both approaches also quantify a different type of evidence in a different fashion.

Summing up, we are dealing with “apples and oranges” when reporting Bayesian and NHST in our study. This fruit salad, however, represents reality in the current scientific literature. The best recommendation might be not to decide for one fruit or the other (e.g., not to “retire” statistical significance in general as stated by Amrhein et al., 2019) (see for example Correia et al., 2019), but to find more meaningful linguistic terms (e.g., replace “statistical significance” by “statistical accuracy,” Correia et al., 2019), to interpret the context of the *p*-value rather than the threshold (Betensky, 2019), and in general to enhance rather than reduce complexity by describing and interpreting data in more detail and nuances (“embrace uncertainty,” this expression was used by Amrhein et al., 2019, p. 32). This is a longer, but instructive process for scientists usually dealing with content-based research questions (as the authors of the present paper) that surely requires patience from statistical experts.

## Data Availability Statement

The raw data supporting the conclusions of this article will be made available by the authors, without undue reservation, to any qualified researcher.

## Ethics Statement

Ethical review and approval was not required for the study on human participants in accordance with the local legislation and institutional requirements. The patients/participants provided their written informed consent to participate in this study.

## Author Contributions

JS, WW, and CD planned the study. JP, RR, JR, and GR conducted the study. JS wrote the first draft of the manuscript. GR, WW, and CD revised and finalized the manuscript. All authors contributed to the article and approved the submitted version.

## Conflict of Interest

GR and CD were employed by the Schmieder Clinics (rehabilitation clinic; Kliniken Schmieder). The remaining authors declare that the research was conducted in the absence of any commercial or financial relationships that could be construed as a potential conflict of interest.

## Footnotes

1 For a taxonomy that differentiates perceived fatigue and performance fatigability (see Kluger et al., 2013).
